# Electrochemical Behavior and Determination of Rutin on Modified Carbon Paste Electrodes

**DOI:** 10.1100/2012/394756

**Published:** 2012-04-30

**Authors:** Pavla Macikova, Vladimir Halouzka, Jan Hrbac, Petr Bartak, Jana Skopalova

**Affiliations:** ^1^Regional Centre of Advanced Technologies and Materials, Department of Analytical Chemistry, Faculty of Science, Palacky University, 17. listopadu 12, 771 46 Olomouc, Czech Republic; ^2^Department of Physical Chemistry, Faculty of Science, Palacky University, 17. listopadu 12, 771 46 Olomouc, Czech Republic; ^3^Department of Analytical Chemistry, Faculty of Science, Palacky University, 17. listopadu 12, 771 46 Olomouc, Czech Republic

## Abstract

The performances of ionic liquid (1-hexyl-3-methylimidazolium-bis(trifluoromethylsulfonyl)imide, IL/CPE) and iron phthalocyanine (IP/CPE) modified carbon paste electrodes in electroanalytical determinations of rutin were evaluated and compared to the performance of unmodified carbon paste electrode (CPE). Cyclic voltammetry (CV), differential pulse voltammetry (DPV), differential pulse adsorptive stripping voltammetry (DPAdSV), and amperometry were used for rutin analysis. The best current responses of rutin were obtained at pH 4.0 for all tested techniques. IL/CPE electrode was found to perform best with DPAdSV technique, where a detection limit (LOD) as low as 5 nmol L^−1^ of rutin was found. On the other hand, IP/CPE showed itself to be an optimum choice for DPV technique, where LOD of 80 nmol L^−1^ was obtained. Analytical applicability of newly prepared electrodes was demonstrated on determination of rutin in the model samples and the extracts of buckwheat seeds. To find an optimum method for buckwheat seeds extraction, a boiling water extraction (BWE), Soxhlet extraction (SE), pressurized solvent extraction (PSE), and supercritical fluid extraction (SFE) were tested.

## 1. Introduction

**Figure 1:**
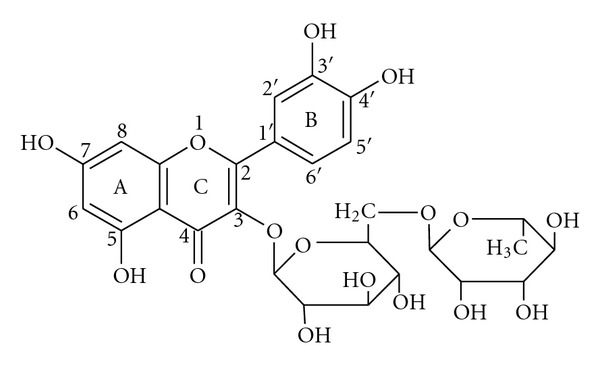
Chemical structure of rutin.

Rutin is a bioactive flavonoid. The structure of rutin (Figure 1) consists of an aglycone quercetin and a disaccharide rutinose bound to quercetin at a position 3, ring C. It has a strong antioxidant activity as proved by different *in vitro* antioxidant assays [1]. Supplementation with rutin increases the total antioxidant status of blood plasma [2, 3].

Rutin is usually determined by high-performance liquid chromatography [4], capillary electrophoresis [5], spectrophotometry [6], and chemiluminescence [7] techniques. Due to the fact that rutin is an electroactive species, electrochemical techniques can also be successfully employed for this task. Electrochemical behaviour of rutin is characterized by two oxidative signals under the conditions similar to the internal environment of human metabolism. The first reversible anodic signal corresponds to two-electron oxidation of –OH groups at positions 3′ and 4′ forming an *o*-quinone. The other irreversible anodic signal is presumably caused by oxidation on the ring A [8].

In the field of electroanalysis, modified carbon paste electrodes (MCPEs) can provide selectivity and sensitivity, resist fouling, concentrate species, improve electroanalytical properties, and limit access of interfering species often present in complex samples or biological fluids. A properly selected modifier can concentrate analyte on an electrode surface or serve as a catalyst of electrochemical reactions [9, 10]. New and still more popular ways of CPE modification are ionic liquids (ILs), which consist of heterocyclic organic cation and various kinds of anions. ILs possess specific physicochemical properties such as excellent ionic conductivity, high chemical and thermal stability, inconsiderable vapor pressure, and wide electrochemical window [11–16].

 Recently, ILs modified carbon paste electrodes have been applied for analysis of rutin. IL-CPEs containing bis(trifluoromethylsulfonyl)imide anion and different imidazolium cations along with laccase from *Aspergillus oryzae* (catalyst of rutin oxidation) allowed determination of micromolar amounts of rutin in pharmaceutical samples [17]. Zhang and Zheng [18] availed an electrocatalytic activity of 1-amyl-3-methylimidazolium bromide modified CPE toward the redox reactions of rutin to its successful quantification in tablets and urine samples. The detection limit of their electrode reached 1 × 10^−8 ^mol L^−1^ of rutin by square wave voltammetry (SWV). Likewise, N-butylpyridinium hexafluorophosphate modified CPE gave strong electrocatalytic effect to the oxidation of rutin [19] and allowed to quantify 3.5 × 10^−7 ^mol L^−1^ of rutin using cyclic voltammetry, a technique far less sensitive than SWV. DNA modified carbon paste electrode containing 1-butyl-3-methylimidazolium hexafluorophosphate ionic liquid and paraffin oil as a binder was used for sensitive detection of rutin [20]. Single-walled carbon nanotubes modified carbon paste electrode with ionic liquid (1-butyl-3-methylimidazolium tetrafluoroborate) was also successfully used for analysis of rutin with similar results [21].

Metals and metallic compounds are often admixed into carbon pastes for example, bismuth [22, 23], gold [24], iron compounds [25–28], manganese (IV) oxide [29, 30], copper (I) oxide [31], and so forth. Iron (II) phthalocyanine as a component of carbon paste electrode has been found to be an effective electrocatalyst of the reduction of organic peroxides [32] and oxidation water in alkaline medium [33]. Recently, it has been also recognized as an efficient electrocatalyst of epinephrine oxidation [34]. Similar electrocatalytic action was reported also for other neurotransmitters containing quinone moiety (dopamine and serotonin) [35]. This finding encouraged us to test the iron (II) phthalocyanine for its electrocatalytical action towards rutin.

In the present work, we prepared state of the art 1-hexyl-3-methylimidazolium-bis(trifluoromethylsulfonyl)imide ([hmim][Tf_2_N]) ionic liquid and iron phthalocyanine modified carbon paste electrodes and tested them in rutin solutions using cyclic voltammetry (CV), differential pulse voltammetry (DPV), differential pulse adsorptive stripping voltammetry (DPAdSV), and amperometry. For comparison purposes, analogical experiments were performed with unmodified carbon paste electrode to evaluate the accessible potential window, the background current, surface reproducibility, and the redox and surface behaviors of rutin. The electrodes were finally applied to rutin determination in extracts from buckwheat seeds (*Fagopyrum esculentum Moench*) by a standard addition method. Four extraction procedures were tested: boiling water extraction (BWE), Soxhlet extraction (SE), pressurized solvent extraction (PSE), and supercritical fluid extraction (SFE) of buckwheat seeds.

## 2. Experimental

### 2.1. Reagents

Carbon pastes were prepared from graphite flakes (Aldrich-Chemie, Steinheim, Germany) and paraffin oil (pharmaceutical grade) or 1-hexyl-3-methylimidazolium-bis(trifluoromethylsulfonyl) imide ([hmim][Tf_2_N]) (≥98.0%, Merck, Darmstadt, Germany). Iron (II) phthalocyanine (≥97%, Fluka Chemie, Buchs, Switzerland) served as carbon paste modifier. Rutin hydrate (≥94%, Sigma-Aldrich Chemie, Steinheim, Germany) was used without further treatment. Standard solution of rutin (1.0 mM) was prepared in methanol (p.a., LachNer, Czech Republic). More diluted solutions were obtained by dilution with redistilled water. Britton-Robinson buffers were prepared from trihydrogen phosphoric acid, acetic acid and trihydrogen boric acid (0.04 M each). Desired pH values were adjusted with sodium hydroxide (0.2 M). Ionic strength of B-R buffers was adjusted to *I* = 0.15 with sodium perchlorate (p.a., Fluka Chemie, Buchs, Switzerland). Acetate buffer was prepared by titration of acetic acid (0.1 M) with sodium hydroxide (0.2 M). All chemicals used to prepare buffers (Lachema, Czech Republic) and acetone (p.a., Penta, Czech Republic) were of analytical grade. Doubly distilled water (Elga, UK) was used in all experiments. Hulled buckwheat seeds were obtained from PROBIO (Czech Republic).

### 2.2. Apparatus

Voltammetric measurements were performed on an Eco-Tribo Polarograph (Polaro-Sensors, Prague, Czech Republic) with Polar 4 software (Polaro-Sensors, Prague, Czech Republic). A three-electrode system involved Ag/AgCl/1 M-KCl reference electrode, a platinum wire auxiliary electrode and carbon paste electrodes were used as working electrodes. When needed, the measured solutions were purged with nitrogen.

Amperometric measurements in stirred solution were done using CHI660 electrochemical workstation (CH Instruments, USA).

UV/VIS spectrophotometer Lambda 25 (Perkin Elmer, USA) was used for the determination of dissociation constant of rutin. pH measurements were done using inoLab 720 (WTW, Germany) pH-meter. A supercritical fluid extractor SEKO-K (SEKO-K, Czech Republic), a pressurized solvent extractor *one *PSE (Applied Separations, USA) and Eppendorf AG 5702 centrifuge (Eppendorf AG, Germany) were used for rutin extractions from buckwheat seeds.

An analytical balance (Kern model ALS 220-4, Kern & Sohn, Balingen, Germany) was used to weigh samples of buckwheat seeds and chemicals for preparation of solutions and carbon pastes.

Comparative measurements of rutin content in buckwheat extracts were performed on an HPLC system Waters (600S Controller), which consisted of a UV/Vis detector (type 486), a pump (type 616), and a 20 *μ*L loop. The detection wavelength was set to 236 nm. The system was operated at room temperature. The analytical column was Tessek (C18, 205 mm × 4.0 mm I.D., 5.0 *μ*m, Separon). The mobile phase consisted of methanol (55%), doubly distilled water (44%), and acetic acid (1%), the flow rate was 0.4 mL/min. The data were collected and evaluated by Clarity software (DataApex, Czech Republic).

### 2.3. The Preparation of Carbon Paste Working Electrodes

The carbon paste was prepared by mixing 200 mg of graphite flakes with 80 *μ*L of paraffin oil. CPE modified with iron phthalocyanine (IP/CPE) was prepared by replacement of 10% (by weight) of graphite flakes by iron (II) phthalocyanine. Ionic liquid CPE (IL/CPE) was prepared from 200 mg graphite and 100 *μ*L [hmim][Tf_2_N]. Each mixture was homogenized in an agate mortar until a cohesive substance was formed. The paste was filled into the teflon electrode body equipped with a piston (inner diameter 2 mm). The electrode surface was renewed before each scan by removing a small amount of paste from the electrode reservoir using a piston and polishing the electrode surface with a smooth paper.

### 2.4. Procedures

Cyclic voltammograms were recorded at scan rate 100 mV s^−1^. DPV experiments were performed at 50 mV pulse amplitude, pulse width was 100 ms, and scan rate 20 mV s^−1^. In both DPV and DPAdSV analysis limits of detection and quantification were determined experimentally as a concentration of rutin, the current response of which three-times (LOD) and ten-times (LOQ) exceeded the signal noise of supporting electrolyte current measured in the potential range of *E*_*p*_ ± 100 mV, where *E*_*p*_ is a peak potential of rutin. The first oxidation peak of rutin was always evaluated. The precision and accuracy of the measurement were evaluated from the model samples of rutin prepared by adding 2 mL, 0.4 mL and 0.06 mL of rutin standard solution (1 mM), respectively, to acetate buffer (pH 4.0) in distilled water of total volume of 1000 mL. A volume of 10 mL was transferred into voltammetric vessel and analyzed using the standard addition method. Three additions of rutin standard solution (0.1 mM) were used. Number of replicated measurements of each model sample was *n* = 5. The HPLC method used for comparative measurement of rutin in model samples and buckwheat extracts was adapted from the literature [36].

Spectrophotometric determination of dissociation constant (p*K*) of rutin was carried out at rutin concentration of 0.2 *μ*M. Ionic strengths of rutin solutions were adjusted to 0.15 M with sodium perchlorate. The p*K* value of rutin was calculated from the measured data using the procedure described in [37].

Amperometric measurements were carried out at constant potential of 600 mV versus Ag/AgCl. The aliquots (50 *μ*L) of rutin standard solution (1 mM) were introduced into electrochemical cell containing 20 mL of supporting electrolyte using a home-made autosampler. Limits of detection were evaluated from calibration curves with QCExpert software (TriloByte, Czech Republic) by the IUPAC recommended direct method of signal [38].

### 2.5. Buckwheat Extracts Preparation

Grinded buckwheat seeds (1 or 2 g weighed with analytical precision) were used to prepare extracts. Boiling water extraction (BWE) was performed by refluxing of the grinded buckwheat seeds for five minutes in 40 mL of double-distilled water. Extract was filled up to 50 mL with double-distilled water. Samples were centrifuged for 20 minutes and then filtered before analysis. Soxhlet extraction (SE) ran with methanol for four hours. This extract was filled up to 50 mL with methanol. Pressurized solvent extraction (PSE) was carried out with acetone at 150 bar pressure and temperature of 100°C. Two 10-minute cycles of static extraction were performed. This extract was filled up to 50 mL with acetone. Supercritical fluid extraction (SFE) with carbon dioxide proceeded under following conditions: pressure 25 MPa, temperature of an extraction cell 35°C, temperature of a restrictor 100°C, temperature of a catch 30°C, time of analysis 30 min and acetone as a solvent.

## 3. Results and Discussion

### 3.1. CV Experiments

**Figure 2:**
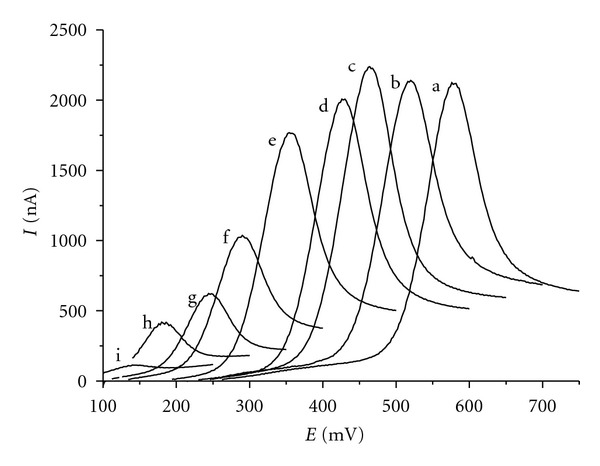
Linear sweep voltammogramms of 0.1 mM rutin in Britton-Robinson buffers measured on CPE at scan rate of 100 mV s^−1^. (a) pH 2, (b) pH 3, (c) pH 4, (d) pH 5, (e) pH 6, (f) pH 7, (g) pH 8, (h) pH 9, (i) pH 9.5.

**Figure 3:**
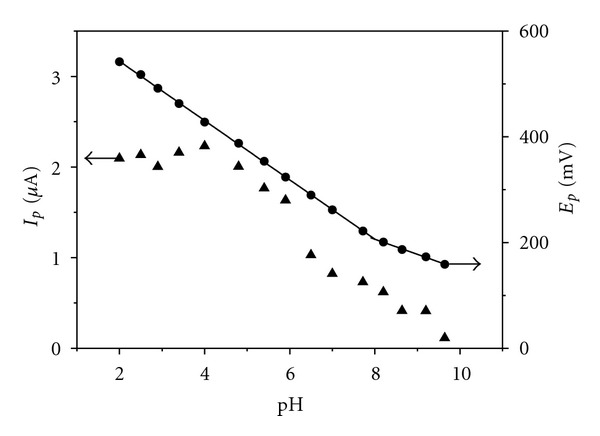
Dependence of anodic CV peak potential and peak current of rutin (*c* = 0.1 mmol L^−1^) on pH. The conditions of the experiments are given in the legend to Figure 2.

As mentioned in Section 1 of this paper, anodic oxidation of rutin proceeds in two subsequent steps. From the point of view of the analytical usability, the first redox transition is more important, therefore further experiments are restricted to this process. In the cyclic voltammogram (Figure 2), the first redox transition manifests itself as a quasireversible, pH-dependent electrode process. The potential of the anodic CV peak shifts to lower values with decreasing acidity (Figure 3). Two regression straight lines fitted to the *E*_*p*_-pH data have slopes of −56 and −29 mV/pH unit. This is consistent with the two-electron oxidation involving the loss of two and one proton, respectively. The intersection point of the two lines at pH 8.0 corresponds to an apparent dissociation constant of rutin. The p*K* value, estimated by us spectrophotometrically at the wavelength of 352 nm to be p*K* = 7.14, is in agreement with the published value of p*K* = 7.1 [39] which is supposed to represent the dissociation of the hydroxyl group in position 4′ of the ring B.

The peak current is maximal in acidic media (pH < 5) and decreases with increasing pH until it falls to zero at pH > 10 (Figure 3). The highest CV peak was obtained at pH = 4, therefore this acidity was chosen for all subsequent experiments.

To characterize the prepared electrodes, initial experiments have been made to compare the accessible potential window and the background charging current. According to expectations the IL/CPE displayed the widest potential window (2.5 V), compared to CPE (1.5 V) and IP/CPE (1.3 V). IL/CPE had also the largest background current (about 60 times higher than that for CPE and IP/CPE). These large background charging currents are typical for electrodes with ionic liquid as a binder [12, 40].

**Figure 4:**
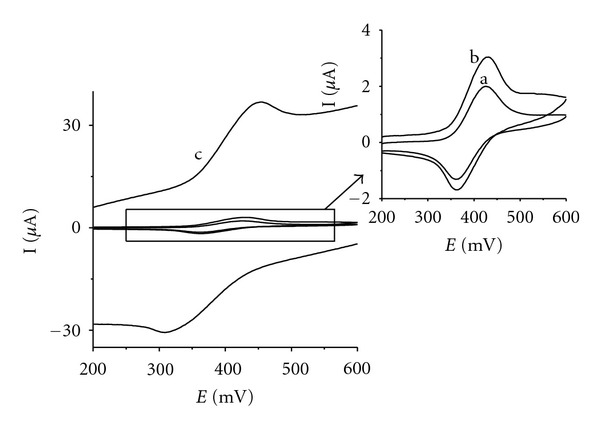
Cyclic voltammograms of rutin on IP/CPE (a), CPE (b), and IL/CPE (c). 0.1 mM rutin solution in acetate buffer (pH 4.0), scan rate 100 mV s^−1^.

All three electrodes gave quasireversible cyclic voltammograms of rutin differing in peak currents and peak separation values (Figure 4, Table 1). The overvoltage for the redox reaction grows at the tested electrodes in the order IP/CPE < CPE ≪ IL/CPE. Simultaneously, the highest ratio of cathodic to anodic peak current demonstrates better reversibility on IP/CPE as compared to CPE and IL/CPE. This means that iron (II) phthalocyanine catalyzed the oxidation of rutin, similarly to other phenols and polyphenols [41, 42]. The peak current of rutin is almost ten-times higher on IL/CPE in comparison to CPE. However, the peak separation inferior to unmodified CPE and a comparable ratio of cathodic to anodic peak indicate that higher current is not caused by electrocatalytic effect of IL/CPE but is rather caused by increased electroactive area of the IL/CPE. This opinion is supported by the fact that background current is increased as well for IL/CPE compared to CPE.

### 3.2. DPV and DPAdSV Experiments

**Figure 5:**
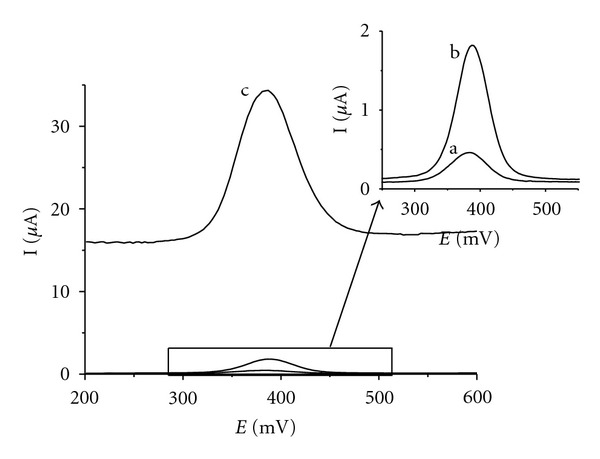
Differential pulse voltammograms of rutin on IP/CPE (a), CPE (b), and IL/CPE (c). 0.008 mM rutin solution in acetate buffer (pH 4.0), scan rate 20 mV s^−1^, pulse amplitude 50 mV, and pulse width 100 ms.

Similarly to CV, the highest DPV peak of rutin was observed on IL/CPE (Figure 5, curve c). Ionic liquid-based electrode provided more than tenfold increase in signal compared to the CPE. Moreover, due to the capability of DPV method to discriminate against the capacitive background current the rutin DPV peaks on IL/CPE are well evaluable and usable for analytical purposes.

**Figure 6:**
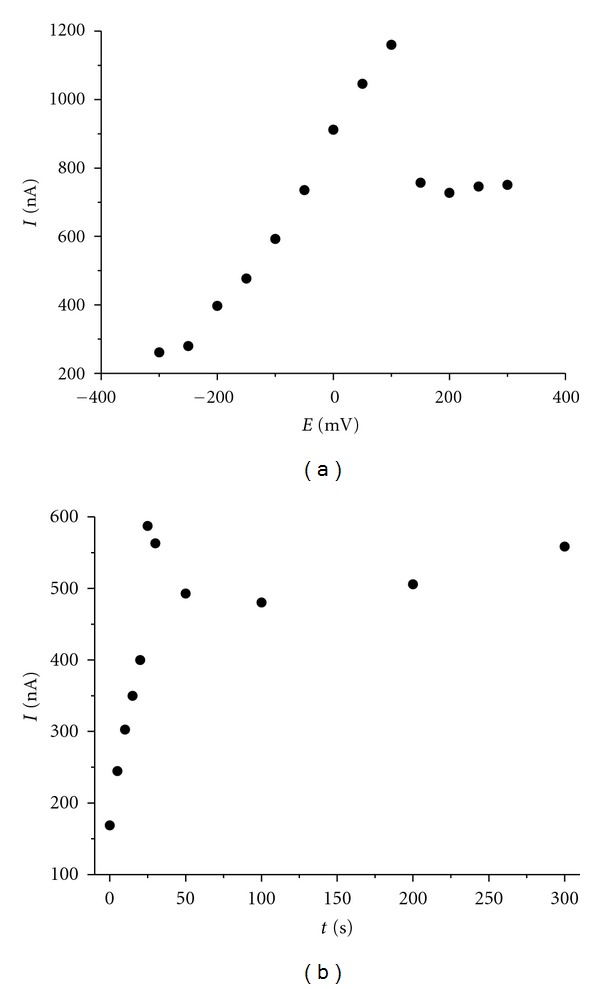
Dependences of DPV peak heights of 2 *μ*M rutin solution in acetate buffer (pH 4.0) at (a) accumulation potential at *t*_acc_ = 25 s and (b) accumulation time (*E*_acc_ = +100 mV). DPV parameters: scan rate 20 mV s^−1^, pulse amplitude 50 mV, and pulse width 100 ms.

We observed a strong adsorption of rutin on all three tested electrodes. First, we tested the adsorption of rutin onto electrode surfaces in an open circuit. The electrodes were dipped into 2 *μ*M rutin solution. After an accumulation period of 5 min, the electrodes were washed with doubly distilled water and placed in an electrochemical cell with supporting electrolyte. A peak of rutin was observed for all electrodes and its height corresponded to 77%, 64%, and 50% of the peak height determined in 2 *μ*M rutin solution on the CPE, IL/CPE and IP/CPE, respectively. No signal of rutin was detected in the supporting electrolyte after renewing surfaces of electrodes. This observation gives the evidence that the electrolyte was not contaminated by the analyte and rutin did not diffuse deeper into the bulk of the electrode material. Subsequently, the dependence of rutin response on accumulation potential and time was examined using DPAdSV. The accumulation in 2 *μ*M rutin solution was the most effective at the potential of +100 mV and time of 25 s (Figure 6). Similar dependences were obtained for all three electrodes.

Calibration dependences of rutin measured by DPV with all three tested electrodes reflect the rutin adsorption. Both DPV and DPAdSV methods give calibration curves linear only in micromolar concentration ranges and nonlinear at higher concentrations. Lower detection limits and narrower linear concentration ranges were typical for DPAdSV. Interestingly, IL/CPE exhibited the best performance in DPAdSV mode, where a very low LOD (5 nmol L^−1^) was found (Table 2). On the other hand, in the case of DPV technique the lowest LOD value was found for IP/CPE (Table 3).

The standard addition method was preferred for the analysis of model samples. Determination of rutin was carried out using both DPV and DPAdSV methods for all three electrodes at three concentration levels (0.06, 0.4, and 2.0 *μ*mol L^−1^of rutin) with three additions of standard solution. Results are summarized in Table 4. All determinations were precise and accurate at the 95% confidence level. Measurement bias was statistically insignificant. Comparative measurements were performed by HPLC-UV/VIS method with 2.0 *μ*M rutin solution measured in five replicates. The relative standard deviation *s*_*r*_ = 4.3% and the percentage bias *B* = 3.5% are comparable to values obtained for voltammetric measurements.

### 3.3. Amperometry

**Figure 7:**
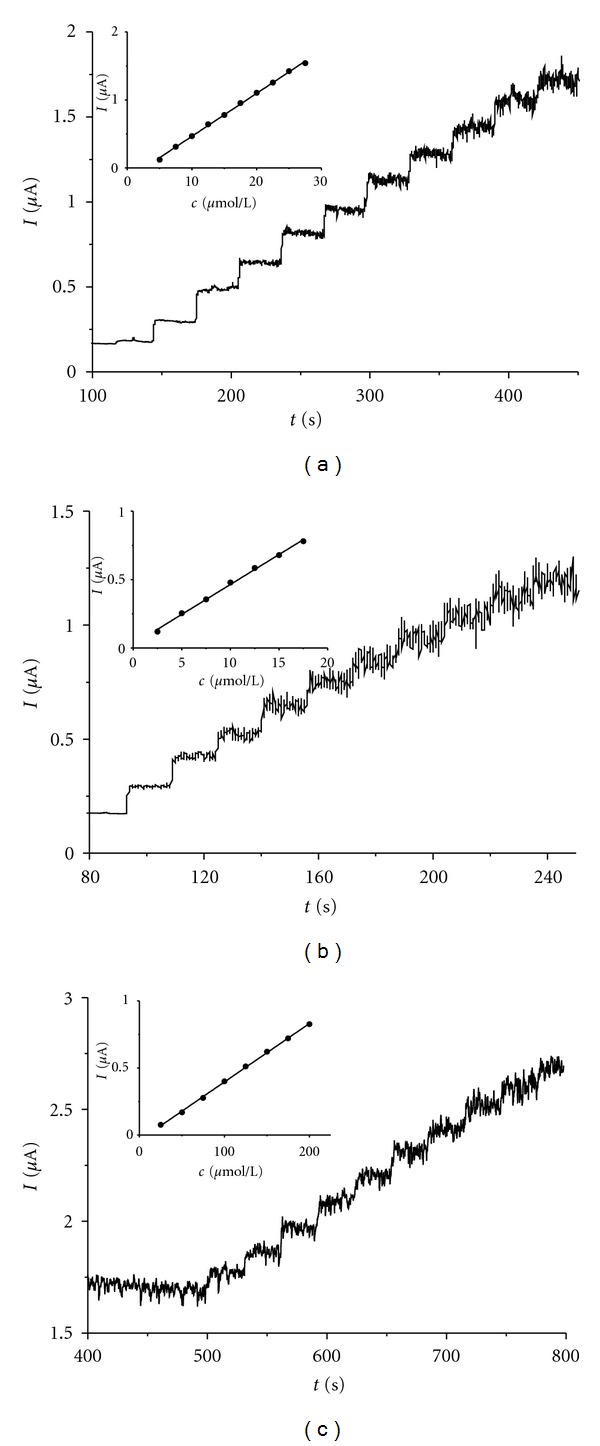
Amperograms and corresponding calibration curves (in insets) of rutin on unmodified (a), iron phthalocyanine modified (b) and ionic liquid modified (c) CPE at 500 mV.

**Figure 8:**
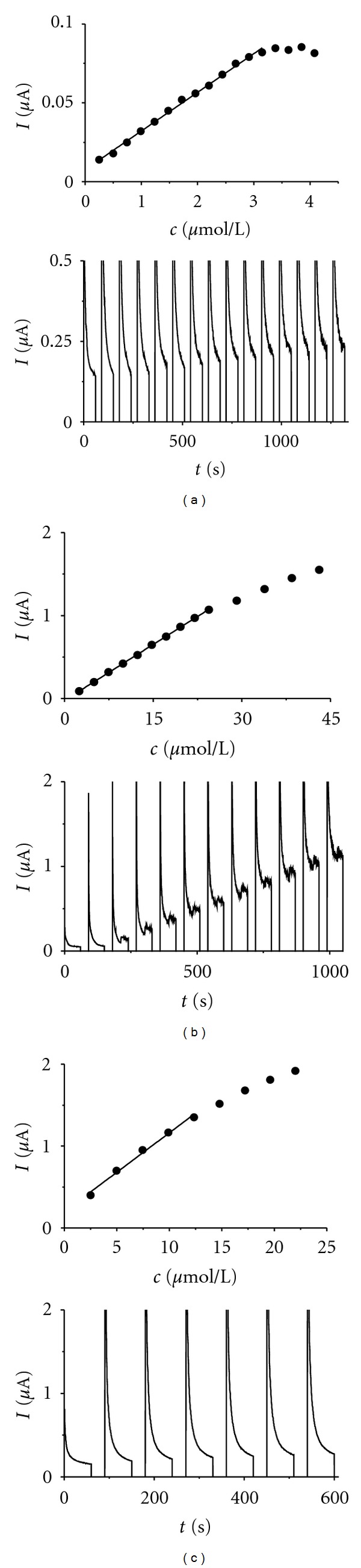
Pulse amperograms (the currents flowing during cleaning step are removed from the amperograms) and corresponding calibration curves of rutin on unmodified (a), iron phthalocyanine modified (b) and ionic liquid modified (c) CPE. The potential was set to +600 mV for 60 s, current was sampled at this point. A 30 s cleaning step at –300 mV followed, during which aliquots of rutin sample solution were introduced into the measuring cell.

After repeated injections of rutin standard solutions an increased noise in the corresponding amperometric response appeared for all electrodes, which may be caused by adsorption of rutin or its oxidation products on the electrode surface (Figure 7). The highest level of noise even in low rutin concentration of 5 *μ*mol L^−1^ exhibited the IL/CPE electrode, which made the amperogram difficult to evaluate. We have found that significant improvement of signal-to-noise ratio is achieved, if pulse technique involving a cleaning step at −300 mV for 30 s is used (Figure 8). The detection limits using the pulsed technique were 0.20 *μ*mol L^−1^ for unmodified CPE, somewhat higher for IP/CPE (0.50 *μ*mol L^−1^) and ten-times higher detection limit was determined for IL/CPE (3.05 *μ*mol L^−1^). The broadest linear concentration range was found for unmodified CPE (0.25–3.1 *μ*mol L^−1^), than IP/CPE (2.5–24.3 *μ*mol L^−1^), and the narrowest one was found for IL/CPE (2.5–12.3 *μ*mol L^−1^), see Figure 8. 

### 3.4. Rutin Determination in Buckwheat Extracts

Four extraction techniques (boiling water extraction, BWE, Soxhlet extraction, SE, pressurized solvent extraction, PSE, and supercritical fluid extraction, SFE) were tested to find an optimum method for isolation of rutin from buckwheat seeds. DPV without accumulation was used as analytical method. No signal of rutin was obtained in extracts from SFE suggesting the used solvent (supercritical CO_2_) is inadvisable for this type of analyte. In PSE extracts, an anodic peak with a potential about 0.1 V lower than that of rutin was observed. This signal corresponded most likely to oxidation of flavonol quercetin as verified by an addition of quercetin standard solution into the measured PSE extract which caused an increase of the peak. The presence of quercetin instead of rutin could be explained either by hydrolysis of rutin to its aglycone or, which is more probable, preferential extraction of less polar quercetin with less polar solvent (acetone). On the other hand, in extracts prepared by BWE and SE only the rutin peak was detected. 

**Figure 9:**
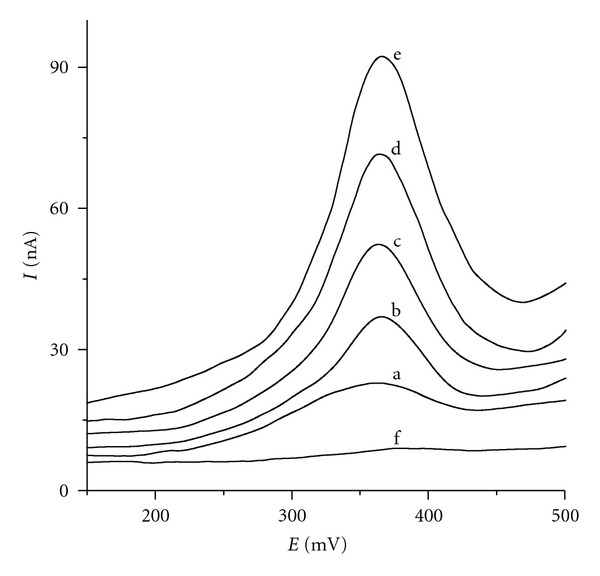
Differential pulse voltammograms of tenfold diluted boiling water extract of buckwheat seeds (a) at IP/CPE and four standard additions of rutin: 20 nmol (b), 40 nmol (c), 60 nmol (d), and 80 nmol (e) in acetate buffer pH = 4 as supporting electrolyte (f).

The quantification of rutin in BWE extract by the method of standard addition (Figure 9) gave 11.8 mg, 12.3 mg, and 11.0 mg of rutin per 100 g of dried buckwheat seeds on CPE, IP/CPE, and IL/CPE, respectively. Comparative measurements by HPLC with UV detection gave the result of 11.4 mg of rutin per 100 g of dried buckwheat seeds. Our results are consistent with the published content of rutin in buckwheat seeds (13.6 mg/100 g, [43]). 

## 4. Conclusions

The electroanalytical behaviour of rutin on two modified CPEs and unmodified CPE was studied in this paper. Iron (II) phthalocyanine as a CPE modifier revealed an electrocatalytic effect on the rutin oxidation. The excellent performance of the carbon paste electrode modified with ionic liquid [hmim][Tf_2_N] was found for rutin determination using DPV technique. Strong adsorption of rutin observed on all electrode materials can be used for sensitivity improvement of voltammetric analysis by DPAdSV. With this technique, limits of detection found with the modified electrodes achieved the nanomolar concentration level. Ionic liquid as a modifier decreased limit of rutin detection in DPAdSV method while iron (II) phthalocyanine lowered LOD in DPV compared to unmodified CPE. Voltammetric methods could be a low-cost and highly sensitive alternative to much more expensive HPLC methods. Noise issues were observed which limit the usability of the studied electrodes to determine rutin by constant potential amperometry in stirred solution. To overcome this problem, a pulse amperometric method was suggested, which achieved a detection limit in submicromolar concentration level of rutin using unmodified CPE. All three studied carbon paste electrodes are usable for analysis of rutin in real samples as has been demonstrated on the analysis of buckwheat seeds extracts.

## Figures and Tables

**Table 1:** Potentials of anodic (*E*_*pa*_) and cathodic (*E*_*pc*_) peak (versus Ag/AgCl, 1 M KCl), peak separation (Δ*E*_*p*_), anodic peak current (*i*_*a*_, the average from seven repeated measurements, standard deviation SD) and ratio of cathodic to anodic peak current (*i*_*c*_/*i*_*a*_) of 0.1 mM rutin at the paraffin/graphite (CPE), paraffin/iron-phthalocyanine/graphite (IP/CPE) and [hmim][Tf_2_N]/graphite (IL/CPE) electrodes (scan rate 100 mV s^−1^).

	*E*_pa_ (mV)	*E*_pc_ (mV)	Δ*E*_*p*_ (mV)	*i*_*a*_ ± SD (*μ*A)	*i*_*c*_/*i*_*a*_
CPE	429	363	66	2.2 ± 0.02	0.65
IP/CPE	427	367	60	1.6 ± 0.02	0.76
IL/CPE	450	318	132	21.5 ± 0.32	0.66

**Table 2:** Parameters of calibration regression straight lines and limits of detection (LOD) and quantification (LOQ) of rutin on the tested carbon paste electrodes, DPAdSV measurement.

Electrode	*c *(*μ*mol L^−1^)	Regression equation *y* = *ax* + *b*	*R*	LOD (mol L^−1^)	LOQ (mol L^−1^)
CPE	0.01–0.1	*y* = 5 × 10^8^ *x* −2.15	0.9784	1 × 10^−8^	3 × 10^−8^
	0.2–1	*y* = 9 × 10^8^ *x* −171.27	0.9886		
IL/CPE	0.005–0.08	*y* = 5 × 10^9^ *x* + 171.95	0.9852	5 × 10^−9^	2 × 10^−8^
IP/CPE	0.02–0.6	*y* = 8 × 10^8^ *x* −13.75	0.9935	2 × 10^−8^	6 × 10^−8^

*c*: linear concentration range, *R:* correlation coefficient.

**Table 3:** Parameters of calibration regression straight lines and limits of detection (LOD) and quantification (LOQ) of rutin on the tested carbon paste electrodes, DPV measurement.

Electrode	*c *(*μ*mol L^−1^)	Regression equation *y *=* ax *+* b *	*R*	LOD (mol L^−1^)	LOQ (mol L^−1^)
CPE	0.2–8	*y* = 2 × 10^8^ *x *−41.90	0.9911	2 × 10^−7^	7 × 10^−7^
IL/CPE	0.6–6	*y* = 3 × 10^8^ *x* + 41.89	0.9944	6 × 10^−7^	2 × 10^−6^
IP/CPE	0.08–6	*y* = 2 × 10^8^ *x *−9.64	0.9955	8 × 10^−8^	3 × 10^−7^

*c:* linear concentration range, *R*: correlation coefficient.

**Table 4:** DPV and DPAdSV determination of rutin in model samples using the method of standard additions. Number of repeated measurements *n* = 5.

Method of measuring	Electrode	Mean content of rutin (*μ*mol L^−1^)	Relative standard deviation *s*_*r*_ (%)	Measurement bias *B* (%)	Recovery *R* (%)
DPV	CPE	1.99	2.6	−0.74	99.3
		0.42	4.3	5.06	105.1
	IL/CPE	2.07	6.3	3.58	103.6
	IP/CPE	2.01	4.7	0.31	100.3
		0.40	4.5	0.98	101.0

DPAdSV	CPE	0.057	4.9	−5.53	94.5
		0.39	3.6	−2.85	97.2
	IL/CPE	0.064	9.1	7.27	107.3
		0.42	6.8	4.10	104.1
	IP/CPE	0.063	5.4	4.50	104.5
		0.41	5.1	1.51	101.5

## References

[1] Yang J, Guo J, Yuan J (2008). In vitro antioxidant properties of rutin. *LWT—Food Science and Technology*.

[2] Florek E, Ignatowicz E, Wrzosek J, Piekoszewski W (2005). Effect of rutin on total antioxidant status of rats exposed to cigarette smoke. *Pharmacological Reports*.

[3] Bojňanská T, Frančáková H, Chlebo P, Vollmannová A (2009). Rutin content in buckwheat enriched bread and influence of its consumption on plasma total antioxidant status. *Czech Journal of Food Sciences*.

[4] Liu Q, Cai W, Shao X (2008). Determination of seven polyphenols in water by high performance liquid chromatography combined with preconcentration. *Talanta*.

[5] Chen G, Zhang H, Ye J (2000). Determination of rutin and quercetin in plants by capillary electrophoresis with electrochemical detection. *Analytica Chimica Acta*.

[6] Hassan HNA, Barsoum BN, Habib IHI (1999). Simultaneous spectrophotometric determination of rutin, quercetin and ascorbic acid in drugs using a Kalman Filter approach. *Journal of Pharmaceutical and Biomedical Analysis*.

[7] Song Z, Hou S (2002). Sensitive determination of sub-nanogram amounts of rutin by its inhibition on chemiluminescence with immobilized reagents. *Talanta*.

[8] Ghica ME, Brett AMO (2005). Electrochemical oxidation of rutin. *Electroanalysis*.

[9] Alkire RC, Kolb DM, Lipkowski J, Ross P (2009). *Chemically Modified Electrodes*.

[10] Kalcher K (1990). Chemically modified carbon paste electrodes in voltammetric analysis. *Electroanal*.

[11] Zhang Y, Zheng JB (2007). Comparative investigation on electrochemical behavior of hydroquinone at carbon ionic liquid electrode, ionic liquid modified carbon paste electrode and carbon paste electrode. *Electrochimica Acta*.

[12] Musameh M, Wang J (2008). Sensitive and stable amperometric measurements at ionic liquid-carbon paste microelectrodes. *Analytica Chimica Acta*.

[13] Maleki N, Safavi A, Tajabadi F (2007). Investigation of the role of ionic liquids in imparting electrocatalytic behavior to carbon paste electrode. *Electroanalysis*.

[14] Sun W, Yang M, Jiao K (2007). Electrocatalytic oxidation of dopamine at an ionic liquid modified carbon paste electrode and its analytical application. *Analytical and Bioanalytical Chemistry*.

[15] Sun W, Yang M, Gao R, Jiao K (2007). Electrochemical determination of ascorbic acid in room temperature ionic liquid BPPF_6_ modified carbon paste electrode. *Electroanalysis*.

[16] Wei D, Ivaska A (2008). Applications of ionic liquids in electrochemical sensors. *Analytica Chimica Acta*.

[17] Franzoi AC, Migowski P, Dupont J, Vieira IC (2009). Development of biosensors containing laccase and imidazolium bis(trifluoromethylsulfonyl)imide ionic liquid for the determination of rutin. *Analytica Chimica Acta*.

[18] Zhang Y, Zheng J (2008). Sensitive voltammetric determination of rutin at an ionic liquid modified carbon paste electrode. *Talanta*.

[19] Sun W, Yang M, Li Y, Jiang Q, Liu S, Jiao K (2008). Electrochemical behavior and determination of rutin on a pyridinium-based ionic liquid modified carbon paste electrode. *Journal of Pharmaceutical and Biomedical Analysis*.

[20] Wang Y, Xiong H, Zhang X, Wang S (2010). Detection of rutin at DNA modified carbon paste electrode based on a mixture of ionic liquid and paraffin oil as a binder. *Microchimica Acta*.

[21] Zhu Z, Sun X, Zhuang X, Zeng Y, Sun W, Huang X (2010). Single-walled carbon nanotubes modified carbon ionic liquid electrode for sensitive electrochemical detection of rutin. *Thin Solid Films*.

[22] Dejmkova H, Zima J, Barek J, Vytras K, Kalcher K, Svancara I (2008). Application of carbon paste electrodes with admixed bismuth powder for the determination of 4-amino-3-nitrophenol. *Sensing in Electroanalysis*.

[23] Svancara I, Baldrianova L, Tesarova E, Vlcek M, Vytras K, Sotiropoulos S, Vytras K, Kalcher K (2007). Microscopic studies with bismuth modified carbon paste electrodes: morphological transformation of bismuth microstructures and related observations. *Sensing in Electroanalysis*.

[24] Švancara I, Vytřas K, Bobrowski A, Kalcher K (2002). Determination of arsenic at a gold-plated carbon paste electrode using constant current stripping analysis. *Talanta*.

[25] Hrbac J, Halouzka V, Zboril R, Papadopoulos K, Triantis T (2007). Carbon electrodes modified by nanoscopic iron(III) oxides to assemble chemical sensors for the hydrogen pėroxide amperometric detection. *Electroanalysis*.

[26] Karyakin AA, Gitelmacher OV, Karyakina EE (1994). A high-sensitive glucose amperometric biosensor based on Prussian Blue modified electrodes. *Analytical Letters*.

[27] Karyakin AA, Karyakina EE, Gorton L (1998). The electrocatalytic activity of Prussian blue in hydrogen peroxide reduction studied using a wall-jet electrode with continuous flow. *Journal of Electroanalytical Chemistry*.

[28] Shahrokhian S, Ghalkhani M, Amini MK (2009). Application of carbon-paste electrode modified with iron phthalocyanine for voltammetric determination of epinephrine in the presence of ascorbic acid and uric acid. *Sensors and Actuators B*.

[29] Schachl K, Alemu H, Kalcher K, Ježkova J, Švancara I, Vytřas K (1997). Amperometric determination of hydrogen peroxide with a manganese dioxide-modified carbon paste electrode using flow injection analysis. *Analyst*.

[30] Beyene NW, Kotzian P, Schachl K (2004). (Bio)sensors based on manganese dioxide-modified carbon substrates: Retrospections, further improvements and applications. *Talanta*.

[31] Dursun Z, Nişli G (2004). Voltammetric behavior of copper(I)oxide modified carbon paste electrode in the presence of cysteine and ascorbic acid. *Talanta*.

[32] Qi X, Baldwin RP (1993). Liquid chromatography and electrochemical detection of organic peroxides by reduction at an iron phthalocyanine chemically modified electrode. *Electroanal*.

[33] Chebotareva N, Nyokong T (1997). First-row transition metal phthalocyanines as catalysts for water electrolysis: a comparative study. *Electrochimica Acta*.

[34] Shahrokhian S, Ghalkhani M, Amini MK (2009). Application of carbon-paste electrode modified with iron phthalocyanine for voltammetric determination of epinephrine in the presence of ascorbic acid and uric acid. *Sensors and Actuators B*.

[35] Patrascu D, David I, David V (2011). Selective voltammetric determination of electroactive neuromodulating species in biological samples using iron(II) phthalocyanine modified multi-wall carbon nanotubes paste electrode. *Sensors and Actuators B*.

[36] Michalkiewicz A, Biesaga M, Pyrzynska K (2008). Solid-phase extraction procedure for determination of phenolic acids and some flavonols in honey. *Journal of Chromatography A*.

[37] Kotoucek M (1975). Spectrophotometry of acid-base properties of gallocyanine methyl-ester and its derivatives. *Collection of Czechoslovak Chemical Communications*.

[38] Currie LA (1995). Nomenclature in evaluation of analytical methods including detection and quantification capabilities (IUPAC Recommendations 1995). *Pure and Applied Chemistry*.

[39] Jovanovic SV, Steenken S, Tosic M, Marjanovic B, Simic MG (1994). Flavonoids as antioxidants. *Journal of the American Chemical Society*.

[40] Kachoosangi RT, Wildgoose GG, Compton RG (2007). Room temperature ionic liquid carbon nanotube paste electrodes: overcoming large capacitive currents using rotating disk electrodes. *Electroanalysis*.

[41] Sorokin AB, Kudrik EV (2011). Phthalocyanine metal complexes: versatile catalysts for selective oxidation and bleaching. *Catalysis Today*.

[42] Zagal JH, Griveau S, Silva JF, Nyokong T, Bedioui F (2010). Metallophthalocyanine-based molecular materials as catalysts for electrochemical reactions. *Coordination Chemistry Reviews*.

[43] Morishita T, Yamaguchi H, Degi K (2007). The contribution of polyphenols to antioxidative activity in common buckwheat and Tartary buckwheat grain. *Plant Production Science*.

